# Body Mass Index Is Better than Other Anthropometric Indices for Identifying Dyslipidemia in Chinese Children with Obesity

**DOI:** 10.1371/journal.pone.0149392

**Published:** 2016-03-10

**Authors:** Yanna Zhu, Zixian Shao, Jin Jing, Jun Ma, Yajun Chen, Xiuhong Li, Wenhan Yang, Li Guo, Yu Jin

**Affiliations:** 1 Department of Maternal and Child Health, School of Public Health, Global Health Institute, Sun Yat-sen University, Guangzhou, China; 2 Institute of Child and Adolescent Health, School of Public Health, Peking University, Peking, China; TNO, NETHERLANDS

## Abstract

**Background:**

Body mass index (BMI), waist circumference (WC), and waist-to-hip ratio (WHR) are used in screening and predicting obesity in adults. However, the best identifier of metabolic complications in children with obesity remains unclear. This study evaluated lipid profile distribution and investigated the best anthropometric parameter in association with lipid disorders in children with obesity.

**Methods:**

A total of 2243 school children aged 7–17 years were enrolled in Guangzhou, China, in 2014. The anthropometric indices and lipid profiles were measured. Dyslipidemia was defined according to the US Guidelines for Cardiovascular Health and Risk Reduction in Children and Adolescents. The association between anthropometry (BMI, WC, and WHR) and lipid profile values was examined using chi-square analysis and discriminant function analysis. Information about demography, physical activity, and dietary intake was provided by the participant children and their parents.

**Results:**

Children aged 10–14 and 15–17 years old generally had higher triglyceride values but lower median concentration of total cholesterol, high-density lipoprotein cholesterol, and low-density lipoprotein cholesterol compared with children aged 7–9 years old (all *P* < 0.001). These lipid parameters fluctuated in children aged 10–14 years old. The combination of age groups, BMI, WC and WHR achieved 65.1% accuracy in determining dyslipidemic disorders. BMI correctly identified 77% of the total dyslipidemic disorders in obese children, which was higher than that by WHR (70.8%) (*P*< 0.05).

**Conclusion:**

The distribution of lipid profiles in Chinese children differed between younger and older age groups, and the tendency of these lipid levels remarkably fluctuated during 10 to 14 years old. BMI had better practical utility in identifying dyslipidemia among school-aged children with obesity compared with other anthropometric measures.

## Introduction

Childhood obesity is a serious public threat because of its various metabolic complications and related risks of cardiovascular diseases (CVDs), such as dyslipidemia, hypertension, and diabetes mellitus, which is already potentially present at an early age [1–3]. Cardiovascular risk factors undergo a slow and sub-clinical evolution and usually include the prevalence of abnormalities in lipid metabolism [4–6]. For example, 3.9% to 9.4% of Chilean children aged 11.4 ± 0.97 years old exhibited a clinical form of dyslipidemia [7]. Likewise, approximately one in every five American youth had an adverse lipid concentration of total cholesterol (TC) or high-density lipoprotein cholesterol (HDL-C) [8]. However, few studies have examined blood lipids levels in children stratified by age and gender in low- and middle-income countries such as China.

Many studies have suggested variations in the ability of body mass index (BMI), waist circumference (WC) and waist-to-hip ratio (WHR) in predicting cardiovascular risk factors in adults, which may differ with ethnicity and age groups [9,10]. Child overweight and obesity are often defined by BMI in both clinical practice and biomedical research instead of WC or WHR. Although BMI remains the most popular obesity measurement tool, its main limitation over WC and WHR is its inability to consider body fat distribution. Given the differences in growth status between children and adults, best anthropometry measure to identify adiposity in children remains unclear.

This study aimed to evaluate the distribution of serum lipid levels in Chinese children stratified by age and gender and to estimate the discriminative abilities of BMI, WC, and WHR in screening the risks of dyslipidemia in children with obesity.

## Material and Method

### Design and Study Subjects

The study was a series of cross-sectional health and nutrition surveys conducted by student constitution and local authorities to provide representative prevalence data of the health conditions of children in Guangzhou, China. The study population finally comprised 2243 children aged 7–17 years old who were randomly selected from elementary and middle schools in Guangzhou City in 2014. All children were asked to complete questionnaires, including information on physical activities and dietary intake, with parents completing the demographic information (birth weight, delivery mode, household income, etc.). Participants who refused to finish the questionnaires or did not have complete anthropometric and demographic data were excluded from this analysis (n = 157). Study protocols were approved by the Ethical Committee of the Peking University. All children and their parents voluntarily signed the informed consents.

### Questionnaire Assessment

The self-reported questionnaires were created based on previously tested and validated questions, including questions of demographics, physical activities (vigorous-intensity activities, moderate-intensity activities, walking and sedentary behavior), and dietary intake (breakfast, sugary drinks, fruit, vegetable, and meat). The participant were asked the following five questions regarding physical activities: “How many days and how often per day did you go on vigorous-intensity activities (running, basketball, football, bodybuilding exercises, etc.), moderate-intensity activities (table tennis, moving something light, dancing, etc.) and walking in the last seven days?” The average outdoor duration per day in the last week was classified into four categories (<1.0, 1–2, 2–4 and >4 h/day). Children were also asked the following: “How many hours did you spend in sedentary behavior (sitting or lying still at school and home, not including sleeping) in the last seven days?” To determine their dietary intake of vegetable, meat, fruit and breakfast, children were asked the following: “How many days did you have breakfast last week and how many servings of each dietary intake did you have last week?” and “How many cups of sugary drinks did you have last week?” One serving of sugar-sweetened beverage was 250 mL.

### Anthropometric Measurements

The anthropometric measurements, including height, weight, hip circumference (HC), WC and blood pressure, of all participants were measured by experienced technicians [11]. Body weight was measured using a scale to the nearest 0.1 kg (Hengxing RGT-140, Jiangsu, PRC), and standing height was measured to the nearest 0.1 cm using a fixed stadiometer (Yilian TZG, Jiangsu, PRC). WC and HC were also measured to the nearest 0.1 cm. BMI and WHR were then calculated. The systolic and diastolic blood pressures of children were measured after 5 min in a seated position using a sphygmomanometer (Yutu XJ1ID, Shanghai, PRC).

### Laboratory Analysis

Venous blood samples were collected from children following an overnight fast for 12 h. Serum was separated by centrifugation at 1500×g for 15 min at 4°C within 2 h and stored at −80°C until testing. Serum levels of triglycerides (TG), low-density lipoprotein cholesterol (LDL-C), TC and HDL-C were examined using commercial colorimetric kits (Biosino Biotechnology Company Ltd, Beijing, China) and an automated analyzer (Hitachi Co Ltd) in accordance with previous document [12]. The inter- and intra- assay coefficients of variation for all measured lipid parameters were less than 5%.

### Definition of Abnormal Lipid Levels and Obesity

The 2011 reference data on serum levels of lipids for children according to the US Guidelines for Cardiovascular Health and Risk Reduction in Children and Adolescents were used to estimate whether the lipid indicators of each child were at a normal stage [13]. However, TG was divided in two definitions based on age. Therefore, the highest borderline TG values for ages 0–9 and 10–19 year old (> 99 and > 129 mg/dL, respectively) were used in this assay in both sexes. In accordance with the guideline of the Working Group on Obesity in China, children with <85th percentile BMI for age were considered as normal weight, those with ≥85th but <95th percentile BMI were considered overweight, and those with ≥95th percentile BMI were considered obese [14]. Subjects with ≥ 95th percentile of WC based on Chinese population percentiles were classified as having abdominal obesity [15]. Male children with WHR of ≥ 0.90 and female children with WHR of ≥ 0.85 were classified as obese [16].

### Statistical Analysis

Statistical analysis was performed using SPSS 21.0. Statistically significant differences in median concentration of all serum lipids (TC, TG, HDL-C and LDL-C) stratified by gender and age groups were determined using Student’s *t*-test and analysis of variance (ANOVA). Discriminant function analysis was performed stepwise to test the ability of independent variables (BMI, WC, WHR, age groups, birth weight, physical activities, and dietary intake) in predicting dyslipidemia or non-dyslipidemia. Sensitivity and specificity were also calculated. The categorical variables (delivery mode, household income, and breakfast) were described in both genders by the proportion of participants falling into each category and were evaluated using Chi-square analysis. This type of analysis was also used to compare the proportion of obese children with each item of dyslipidemia in different anthropometric groups. A *P* value of < 0.05 was considered significant.

## Results

### Baseline Characteristics of Study Population

A total of 2243 children with age ranging from 7 to 17 years old were enrolled in this study. The characteristics of the study population, such as demography, lipid profiles, anthropometry, physical activities, and dietary intake, were summarized in Table 1. In terms of lipid profile, boys had significantly lower median concentration of TC, TG, and LDL-C compared with girls (*P* < 0.05). Boys presented higher values of birth weight and anthropometry than girls (*P* < 0.001), except for HC (*P* = 0.437). After comparing the duration of different intensity activities, only vigorous-intensity activities and walking behavior reached a statistical significance between two genders, with *P* < 0.001 and *P* = 0.021, respectively. In addition, consumption of sugary drinks and meat was higher in boys than in girls (*P* < 0.001). Other factors in this study did not significantly differed between boys and girls.

**Table 1:** Descriptive Characteristics of Children Ranging from 7 to 17 Years Old in Guangzhou, 2014 (*n* = 2243).

	Boys	Girls	Total	P-value*
**Demography**				
Sample size (*n*), %	1118 (49.8)	1125 (50.2)	2243 (100)	
Age^a^ (years)	10.95 (± 3.16)	10.66 (± 3.05)	10.80 (± 3.11)	0.03
Birth Weight^a^ (g)	3308.7 (± 463.1)	3203.0 (± 470.8)	3254.7 (± 429.9)	< 0.001
Delivery Mode (*n*), %				0.26
Natural Delivery	519 (23.1)	518 (23.1)	1037 (46.2)	
Cesarean Delivery	437 (19.5)	483 (21.5)	920 (41.0)	
Unknown	162 (7.2)	124 (5.5)	286 (12.8)	
Household Income (*n*), %				0.338
≤ 2000 CNY/month	22 (0.98)	17 (0.76)	39 (1.74)	
~ 5000 CNY/month	109 (4.86)	118 (5.26)	227 (10.12)	
~ 8000 CNY/month	146 (6.52)	171 (7.62)	317 (14.14)	
Unknown	841 (37.49)	819 (36.51)	1660 (74.00)	
**Anthropometry**				
Height (cm)	145.0 (133.0, 166.5)	145.1 (131.7, 158.3)	145.1 (132.5, 160.7)	< 0.001
Weight (kg)	40.4 (28.4, 54.9)	36.5 (26.2, 47.0)	38.3 (27.3, 50.3)	< 0.001
Body Mass Index (BMI) (kg/m^2^)	17.86 (15.57, 20.71)	16.79 (14.79, 19.19)	17.30 (15.17, 19.85)	< 0.001
Waist Circumference (WC) (cm)	64.2 (71.8, 84.9)	62.3 (56.0, 69.0)	63.0 (56.0, 70.0)	< 0.001
Hip Circumference (HC)(cm)	75.8 (65.1, 84.9)	75.5 (65.0, 84.3)	75.8 (65.0, 84.6)	0.437
Waist-Hip Ratio (WHR)	0.86 (0.82, 0.90)	0.84 (0.80, 0.88)	0.81 (0.85, 0.89)	< 0.001
Systolic Blood Pressure (mmHg)	99.00 (91.00, 105.00)	94.00 (89.00, 100.00)	97.00 (90.00, 101.50)	< 0.001
Diastolic Blood Pressure (mmHg)	61.00 (59.00, 67.00)	60.00 (57.00, 64.00)	61.00 (58.00, 66.00)	< 0.001
**Lipid profile**				
Total Cholesterol (TC) (mmol/L)	4.14 (3.71, 4.68)	4.26 (3.82, 4.76)	4.20 (3.67, 4.71)	0.002
Triglyceride (TG) (mmol/L)	0.76 (0.59, 0.98)	0.80 (0.63, 0.80)	0.78 (0.61, 1.03)	0.001
HDL Cholesterol (mmol/L)	1.35 (1.16, 1.56)	1.38 (1.19, 1.59)	1.36 (1.17, 1.57)	0.132
LDL Cholesterol (mmol/L)	2.24 (1.86, 2.69)	2.31 (1.92, 2.77)	2.28 (1.89, 2.75)	0.04
**Physical activities time spent, median**				
Vigorous-Intensity Activities (minute/d)	140.0 (60.0, 315.0)	90.0 (20.0, 200.0)	120.0 (40.0, 240.0)	< 0.001
Moderate-Intensity Activities (minute/d)	120.0 (40.0, 240.0)	100.0 (40.0, 210.0)	105.0 (40.0, 210.0)	0.206
Walking (minute/d)	210.0 (120.0, 420.0)	210.0 (116.3, 420.0)	210.0 (120.0, 420.0)	0.021
Sedentary Behavior Time Spent (hour/week)	38.5 (14.0, 56.0)	37.9 (14.0, 56.0)	38.5 (14.0, 56.0)	0.853
**Dietary intake**				
Sugary Drink Consumption (mL/week)	250.0 (0.0, 1000.0)	250.0 (0.0, 750.0)	250.0 (0.0, 750.0)	< 0.001
Breakfast Intake Every Day (*n*), %	910 (40.57)	958 (42.71)	1868 (83.28)	0.086
Fruit (servings/week)	7.0 (4.0, 12.0)	7.0 (5.0, 12.0)	7.0 (4.0, 12.0)	0.181
Vegetable (servings/week)	14.0 (7.0, 14.0)	14.0 (7.0, 14.0)	14.0 (7.0, 14.0)	0.662
Meat (servings/week)	7.0 (7.0, 14.0)	7.0 (7.0, 14.0)	7.0 (7.0, 14.0)	< 0.001

^a^Mean (± standard deviation) presented

Median (Interquartile range) presented unless otherwise stated. HDL, high-density lipoprotein; LDL, low-density lipoprotein

*P-value was calculated using chi-square test for categorical variable and two independent samples *t-*test for continuous variable between genders.

### Distribution of Lipid Profile in Children Stratified by Sex and Age

As shown in Table 2, the distribution of serum lipids of the sample substantially varied and all indicators demonstrated markedly significant differences among ages based on ANOVA method (all *P* < 0.001). To further analyze the influence of age groups on lipid profiles, we divided ages into three groups: 7–9, 10–14 and 15–17 year old. We then found that compared with children aged 7–9 years old, the two older age groups tended to have lower median concentration of TC, HDL-C, and LDL-C and higher TG (*P* < 0.05).

**Table 2:** Median Concentration (IQR) of the Lipid Profile among Children Aged 7–17 Years in This Study (*n* = 2243).

Age(y)	TG (mmol/L)^a^^b^	TC (mmol/L)^a^^b^	HDL-C (mmol/L)^a^^b^	LDL-C (mmol/L)^a^^b^
7–	0.69 (0.56, 0.85)	4.33 (3.85, 4.76)	1.43 (1.25, 1.66)	2.33 (1.94, 2.80)
8–	0.72 (0.56, 0.89)	4.29 (3.85, 4.87)	1.45 (1.24, 1.67)	2.33 (1.97, 2.80)
9–	0.74 (0.57, 0.96)	4.44 (3.99, 4.95)	1.42 (1.25, 1.61)	2.51 (2.15, 2.93)
10–	0.81 (0.62, 1.10)	4.43 (4.01, 4.98)	1.42 (1.25, 1.62)	2.49 (2.07, 3.00)
11–	0.77 (0.59, 1.03)	4.02 (3.44, 4.70)	1.39 (1.21, 1.55)	2.12 (1.76, 2.94)
12–	0.96 (0.72, 1.24)	4.05 (3.65, 4.55)	1.30 (1.15, 1.50)	2.10 (1.73, 2.43)
13–	0.79 (0.65, 1.05)	4.00 (3.62, 4.44)	1.27 (1.14, 1.49)	2.12 (1.81, 2.46)
14–	0.82 (0.65, 0.95)	3.81 (3.41, 4.23)	1.18 (1.03, 1.38)	2.10 (1.68, 2.41)
15–	0.85 (0.69, 1.13)	4.02 (3.63, 4.53)	1.27 (1.10, 1.46)	2.20 (1.84, 2.59)
16–	0.87 (0.70, 1.10)	4.02 (3.60, 4.42)	1.24 (1.10, 1.45)	2.15 (1.83, 2.56)
17–	0.89 (0.61, 1.05)	3.94 (3.67, 4.60)	1.34 (1.13, 1.49)	2.07 (1.79, 2.66)
7–9	0.71 (0.57, 0.90)	4.35 (3.90, 4.83)	1.43 (1.25, 1.65)	2.38 (1.99, 2.82)
10–14	0.84 (0.66, 1.13)^c^	4.13 (3.73, 4.66)^c^	1.33 (1.16, 1.53)^c^	2.23 (1.86, 2.67)^c^
15–17	0.85 (0.69, 1.10)^c^	4.02 (3.61, 4.48)^c^^d^	1.26 (1.11, 1.46)^c^^d^	2.18 (1.81, 2.59)^c^

HDL-C, high-density lipoprotein cholesterol; LDL-C, low-density lipoprotein cholesterol; TC, total cholesterol; TG, triglyceride. Median (interquartile range) presented unless otherwise stated.

^a^ Significant levels by ANOVA among ages.

^b^ Significant levels by ANOVA among three age groups.

^c^ Significant differences between age groups using *t*-test analysis (7–9-year old group as reference category).

^d^ Significant differences between age groups using *t*-test analysis (10–14-year old group as reference category).

**Fig 1:**
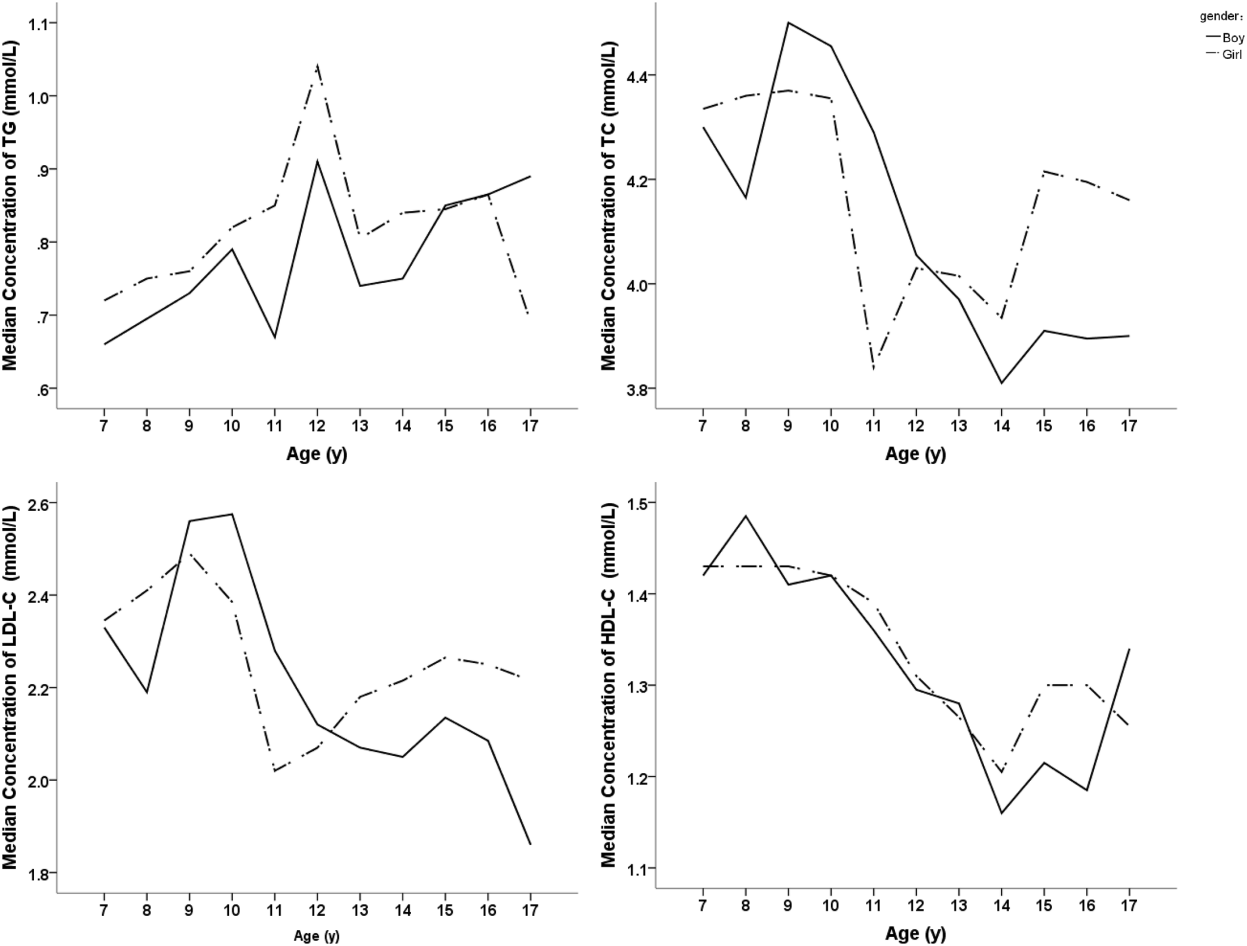
Distribution of Lipid Profile in Boys and Girls Aged 7–17 Years Old (*n* = 2243).

We further explored each lipid profile item to evaluate whether the trends observed in the total lipid profile were generalized by sex (Fig 1). In children aged 10–14 years old, the TC, HDL-C, and LDL-C levels decreased with the increase in ages in both genders and declined to a valley at 14 years (Fig 1B, 1C and 1D). The tendency of TG fluctuated intensively from 10 to 14 years old and reached a peak level at 12 years old (Fig 1A).

### Variables to Predict Dyslipidemia in Children Determined by Discriminant Function Analysis

We involved different variables (BMI, WC, WHR, age group, birth weight, physical activities, and dietary intake) in the discriminant function analysis to analyze which variable contributed to predicting dyslipidemia. Table 3 showed that a combination of four variables (BMI, WC, WHR, and age groups) was selected to correctly classify children into the dyslipidemia or non-dyslipidemia groups. The model with the BMI alone correctly classified 67.9% of the dyslipidemia better than the other three variables (WC, WHR, and age groups).

**Table 3:** Results of Discriminant Function Analysis for Classifying Dyslipidemia among Children (*n* = 2243).

Variables	Standardized Discriminant Function Coefficients	Accuracy	Sensitivity	Specificity
Age groups (y)	0.445	53.6%	56.3%	47.5%
BMI (kg/m^2^)	0.484	67.9%	85.0%	28.8%
WC (cm)	0.396	67.2%	84.3%	28.2%
WHR	0.397	54.0%	51.8%	58.9%

Formula: Z = 0.445 age groups (y) +0.484 BMI (kg/m^2^) + 0.396 WC (cm) +0.397 WHR

BMI, body mass index; WHR, waist to hip ratio; WC, waist circumference.

### Percentage of Dyslipidemia in Children with Obesity as Defined by BMI, WC and WHR

The proportion of children with dyslipidemia was shown in Table 4. Based on the established cut-off points, the proportion of high TG and LDL-C in obese children group defined by the combination of BMI, WC, and WHR was higher than those in other groups (*P* < 0.001), whereas the combination of BMI and WC had the highest proportion of high TC and low HDL-C (*P* < 0.05). After evaluating the number of dyslipidemic disorders, over 70.8% of obese subjects had at least one risk factor (high TG, high TC, high LDL-C, or low HDL-C). Among obese children, the percentage of having all four cardiovascular risk factors was between 3.6% and 8.9% by all six measures of obesity, with the highest proportion for those defined by the combination of BMI, WC, and WHR. In addition, the percentage for dyslipidemic disorders in children with obesity was higher as defined by BMI (77%) than that as defined by WHR (70.8%), with *P* < 0.05.

**Table 4:** Proportion of Lipid Disorders in Children Defined by BMI, WC, and WHR (*n* = 2243).

	Obesity (as defined by)						P-value ^a^
	BMI (*n* = 177)	WC (*n* = 431)	WHR (*n* = 1154)	BMI + WC (*n* = 171)	WC + WHR (*n* = 360)	BMI + WC + WHR (*n* = 146)	
**Dyslipidemia (*n*), %**							
High TG	96 (54.2)	197 (45.7)	403 (34.9)**	95 (55.6)	170 (47.2)	84 (57.5)	< 0.001
High TC	97 (54.8)	189 (43.9)*	525 (45.5)*	94 (55.0)	161 (44.7)*	78 (53.4)	0.015
Low HDL-C	65 (36.7)	154 (35.7)	270 (23.4)**	65 (38.0)	123 (34.2)	54 (37.0)	< 0.001
High LDL-C	57 (32.2)	116 (26.9)	299 (25.9)	55 (32.2)	106 (29.4)	48 (32.9)	0.163
**Number of dyslipidemic factors (*n*), %**							
1 ^#^	138 (77%)	326 (75.6%)	817 (70.8%)*	144 (84.2%)	272 (75.6%)	120 (82.2%)	0.003
2	101 (57%)	223 (51.7%)	481 (41.7%) **	107 (62.6%)	194 (53.9%)	92 (63.0%)	0.081
3	45 (25.4%)	86 (20.0%)	157 (13.6%)	45 (26.3%)	74 (20.6%)	39 (26.7%)	< 0.001
4	13 (7.3%)	21 (4.9%)	42 (3.6%)*	13 (7.6%)	20 (5.6%)	13 (8.9%)	0.016

HDL-C, high-density lipoprotein cholesterol; LDL-C, low-density lipoprotein cholesterol; TC, total cholesterol; TG, triglyceride; BMI, body mass index; WHR, waist to hip ratio; WC, waist circumference.

* *p* < 0.05 level

** *p* < 0.01 level

^#^ The proportion of children with obesity who had at least one risk factor of dyslipidemia (high TG, high TC, high LDL-C, or low HDL-C).

^a^ *P*-value was calculated using chi-square test for category variables. These tests were done to compare among all groups.

## Discussion

With the rapidly increasing prevalence of obesity among children in recent years, a great interest has been given in exploring the relationship between bodily stoutness and blood lipids in children [1,2]. BMI, WC, and WHR were often used to define obesity. However, whether these anthropometric parameters can be used to identify obesity-related complications has not been thoroughly examined. The present study found that the distribution of serum lipid levels in younger groups (aged 7–9 years old) was significantly different from those in other two older age groups (10–14 and 15–17 year old) among Chinese children. Furthermore, the tendency of these lipid parameters remarkably fluctuated in children aged 10–14 years old. Four variables were selected (age groups, BMI, WC, and WHR) via stepwise discriminant procedure and produced 65.1% accuracy in dyslipidemia determination. BMI was better in predicting dyslipidemia in children with obesity compared with WC and WHR.

Yip *et al*. demonstrated that lipid levels, such as TC, LDL-C, HDL-C, and apo B, showed gender-related differences among 525 subjects in early school and adolescent ages [17]. These results are consistent with the present finding that boys have significantly lower median concentration of TC, TG, and LDL-C than girls. In addition, we found that a sharp decrease in TC, HDL-C, and LDL-C, whereas the tendency of TG intensively fluctuated during 10–14 years. The fluctuation in TG in children aged 10–14 years may be attributed to their transitioning from pre-pubertal to pubertal status. Previous studies among school-aged children proposed that the significant reductions in the levels of lipid profiles were associated with the hormones produced during puberty [18,19]. However, a retrospective analysis reported that no differences in lipids were found in 43 girls treated with gonadotropin-releasing hormone analog for at least 2 years compared with those untreated during 3-year follow-up [20]. Together with other research groups, we speculate that the girls had a relatively protective tendency when they entered the puberty years [21]. The role of hormone on levels of lipid profiles during puberty may depend, to some extent, on the duration of observation and the study population. Thus, the relationship between lipids and hormonal levels needs to be further explored in a larger population-based longitudinal study, which can be useful in tracking changes in lipids during the maturation process.

As reported previously, children with overweight and obesity are more likely to experience dyslipidemia and related CVD risk [22–26]. A case-control study observed that children with obesity aged 7–10 years old defined by BMI had 2.17 times greater risks of high TC and hypertension than non-obese children [23]. Another study reported that obesity, rather than being overweight, was more positively correlated with adverse alterations in the lipid profiles in children [25]. Some previous studies have showed that BMI, WC, and WHR were independent predictors of cardiovascular risk factors, such as in predicting dyslipidemia [9,27]. However, a recent study conducted in children and adolescents demonstrates that WC and WHR are practical measures of abdominal fat mass and are related to increased muscle or distribution of excess fat in the body [28]. Therefore, measures of adiposity need to be further examined to determine the ability of these measures to identify children at the risk of dyslipidemia. To elucidate this point, the present study confirmed that BMI, instead of WC or WHR, produced a better role in correctly classifying children into dyslipidemia or non-dyslipidemia groups by using stepwise disciminant analysis.

Furthermore, this study conducted among Chinese children showed that a combination of BMI, WC, and WHR yielded the highest proportion for those with one of the lipid disorders. Adiposity greatly contributed to adverse HDL-C and TG levels, which can be explained by visceral fat accumulation and excess liver exposure to fatty acids [29]. Although more children were identified with at least one dyslipidemic risk factors using the combination of obesity indices (BMI, WC, and WHR) than BMI alone, no statistical significance was found between these two anthropometric groups. In additon, measures of central adiposity (WC and/or WHR) appeared to identify dyslipidemia in older individuals [10]. In accordance with the previous study, WC and WHR were similarly performed after adjustment for age [30]. The present study proposed that BMI may be a better and appropriate risk predictor of dyslipidemia in Chinese children than other anthropometric indices.

### Strengths and Limitations

This study had several strengths. First, a cluster of potential risk factors, such as birth weight, physical activities, and dietary intake, were considered when exploring the relationship between BMI and CVD. Second, this study involved a wide rang of ages from 7 to 17 years to observe the secular trend of lipid profile. We found that lipid levels tended to remarkably fluctuate in children aged 10–14 years old. Therefore, the participants were divided into three age groups to further explore the relationship between anthropometric parameters and lipid profile values. However, this study also had limitations. A cross-sectional design was applied in this study, which cannot infer the causality of the relationships observed. Moreover, we were unable to conclude on the role of pubertal timing on the variations in serum lipids in this age group, as pubertal stage was not assessed in this population

## Conclusion

We found different distributions of serum lipid levels among three age groups (7–9, 10–14, and 15–17 years old) in Chinese children and found significant fluctuations among these lipid parameters in children aged 10–14 years old. This study proved that BMI can be used as a simple and non-invasive method in predicting dyslipidemia in Chinese school-aged children with obesity compared with other anthropometric indices.

## Supporting Information

S1 Dataset Dataset of Body Mass Index Is Better than Other Anthropometric Indices for Identifying Dyslipidemia in Chinese Children with Obesity. (XLS)
